# Cytotoxic Effects of Flowable Composite Resins with Different Resin Matrix Compositions on L929 Fibroblasts

**DOI:** 10.3290/j.ohpd.c_2787

**Published:** 2026-08-26

**Authors:** Sema Yazıcı Akbıyık, Cansu Yikici Col, Songul Kilic, Demet Kacaroglu

**Affiliations:** a Sema Yazıcı Akbıyık Assistant Professor, Department of Restorative Dentistry, Faculty of Dentistry, Lokman Hekim University, Ankara, Turkey; wrote the original draft, was involved in the conceptualisation of the study, methodology, investigation, validation, formal analysis, resources, data curation, and visualisation, and reviewed and edited the manuscript; b Cansu Yikici Col Expert, Panorama Ankara Oral and Dental Health Polyclinic, Ankara, Turkey; performed the investigation, reviewed and edited the manuscript, and was involved in the conceptualisation of the study, methodology, resources, and visualisation; c; d Songul Kilic Assistant Professor, Department of Restorative Dentistry, Faculty of Dentistry, Lokman Hekim University, Ankara, Turkey; reviewed and edited the manuscript and was involved in the conceptualisation of the study, resources, and visualisation; e Demet Kacaroglu Assistant Professor, Medical Biology Department, Faculty of Medicine, Lokman Hekim University, Ankara, Turkey; was involved with the methodology, software, formal analysis, resources, and data curation and reviewed and edited the manuscript

**Keywords:** cell viability, cytotoxicity, flowable composite resins, L929 fibroblast, MTT assay

## Abstract

**Objective:**

To evaluate the cytotoxic effects of flowable composite resins with different resin matrix characteristics on L929 fibroblast cells.

**Materials and methods:**

Five flowable composite resins (GrandioSO Flow [GSF; VOCO Dental, Cuxhaven, Germany], EverX Flow [EXF; GC Europe, Leuven, Belgium], Admira Fusion Flow [AFF; VOCO Dental], Stark Bulk Fill Flow [SBFF; President Dental, Allershausen, Germany], and Estelite Bulk Fill Flow [EBFF; Tokuyama Dental, Tokyo, Japan]) were prepared as standardised discs (5 × 2 mm). Extracts obtained after 1 and 7 days of incubation were applied to L929 fibroblast cultures at different concentrations (direct, 1:2, 1:5, and 1:10 dilutions) for 24 and 48 hours. Cell viability was assessed using the 3-(4,5-dimethylthiazol-2-yl)-2,5 diphenyl tetrazolium bromide (MTT) assay. Data were analysed using a two-way analysis of variance (ANOVA) and Tukey post hoc test (α = 0.05).

**Results:**

Direct exposure to all material extracts significantly reduced cell viability compared with the control group (*P* < 0.0001). Cell viability increased with increasing dilution for all materials. EBFF and EXF consistently demonstrated the highest viability values and the most favourable biocompatibility profiles. In contrast, GSF exhibited the lowest cell viability values and the highest cytotoxic potential, particularly under direct exposure. AFF and SBFF showed intermediate responses. Overall, cell viability values were generally higher after 48 hours of exposure than after 24 hours.

**Conclusion:**

The cytotoxic effects of the flowable composite resins were concentration- and time-dependent. Although all tested materials generally maintained satisfactory cell viability under the evaluated conditions, significant differences were observed among the investigated materials. EBFF and EXF exhibited the most favourable biocompatibility, whereas GSF showed comparatively lower cell viability. Resin matrix characteristics, together with polymerisation-related factors, may influence the biological response of flowable composite resins.

Dental composite resins are among the most widely used restorative materials in contemporary dentistry because of their favourable mechanical and esthetic properties.^28^ Ideally, their mechanical behaviour, wear resistance, and optical characteristics should closely resemble those of natural dental tissues.^30^ Despite continuous improvements in material formulations, challenges such as polymerisation shrinkage, cytotoxicity, microleakage, secondary caries, and restoration fracture remain important clinical concerns.^9,12,13,14,28
^


Composite resins are composed of inorganic filler particles embedded within an organic resin matrix and coupled through an interfacial phase.^8,29
^ The resin matrix typically contains monomers such as bisphenol A glycidyl methacrylate (Bis-GMA), urethane dimethacrylate (UDMA), triethylene glycol dimethacrylate (TEGDMA), 2-hydroxyethyl methacrylate (HEMA), and ethoxylated bisphenol A dimethacrylate (EBPDMA). The biological behaviour of resin-based materials is largely influenced by the characteristics of the resin matrix, including monomer composition, molecular structure, molecular weight, and the potential for diffusion of residual components.^1,27
^


Flowable composite resins were originally considered unsuitable for stress-bearing areas because of their relatively low filler content. However, advances in filler technology and resin matrix formulations have significantly improved their mechanical performance while reducing polymerisation shrinkage, thereby expanding their clinical applications.^19^ Conventional flowable composites are typically placed using an incremental technique at a thickness of 2 mm due to the limited depth of polymerisation and to reduce polymerisation shrinkage.^15^ However, this technique is time-consuming and may increase the risk of air entrapment or contamination between layers.^30^ To overcome these limitations, bulk-fill flowable composites were developed, allowing placement in thicker layers through enhanced translucency and modified resin matrix systems that improve curing efficiency and reduce shrinkage stress.^29^


To improve biocompatibility and reduce polymerisation-related stress, composite resins incorporating modified organic matrix systems have been developed.^15^ Among these materials, ormocer-based composites (organically modified ceramics) have attracted considerable interest because of their reduced polymerisation shrinkage, favourable wear resistance, and promising biological properties.^16,29
^ In addition, their coefficient of thermal expansion has been reported to be closer to that of natural tooth structure than that of conventional resin composites.^16^


In recent years, short fibre–reinforced composite resins have been developed to enhance the mechanical performance of restorative materials.^29^ These materials generally exhibit greater fracture resistance than conventional composite resins and are particularly suitable for stress-bearing areas, such as posterior restorations exposed to high occlusal loads.^11,29
^ In addition to improving mechanical properties, fibre reinforcement may help reduce microleakage by promoting a more favourable distribution of polymerisation shrinkage stresses.^11^


The cytotoxicity of resin-based materials is primarily associated with the release of residual monomers resulting from incomplete polymerisation.^10^ Furthermore, other components, including polymerisation initiators present in the organic matrix and metal ions released from inorganic filler particles, may also contribute to their cytotoxic effects.^7,10
^


Although the cytotoxicity of conventional and bulk-fill composite resins has been investigated extensively, studies directly comparing flowable composite resins with different resin matrix characteristics under standardised conditions remain limited.^1,15,28
^ As such, this study aimed to evaluate and compare the cytotoxic effects of selected flowable composite resins on L929 mouse fibroblasts. The null hypothesis tested was that no significant differences would be observed in the cytotoxic responses induced by the investigated materials.

## Materials and methods

Five flowable composite resins (GrandioSO Flow [GSF; VOCO Dental, Cuxhaven, Germany], EverX Flow [EXF; GC Europe, Leuven, Belgium], Admira Fusion Flow [AFF; VOCO Dental], Stark Bulk Fill Flow [SBFF; President Dental, Allershausen, Germany], and Estelite Bulk Fill Flow [EBFF; Tokuyama Dental, Tokyo, Japan]) were evaluated in this study. The characteristics and compositions of the tested materials are presented in Table 1. Information regarding resin matrix composition and filler content was obtained from the manufacturers’ safety data sheets, instructions for use, and technical documentation.

**Table 1 Table1:** Materials used in the study

Material	Lot number	Curing time	Organic matrix (monomers)	Inorganic filler
GrandioSO Light Flow (GSF)	2423169	20 sec	HEDMA, BisGMA, TEGDMA, BisEMA	Barium aluminium borosilicate glass, silicon dioxide, fumed silica, total ≈76% w/w
EverX Flow (EXF)	2409251	10 sec	Bis-MEPP, TEGDMA, UDMA	Short E-glass fibres (~25% w/w) + barium glass fillers (~42–52% w/w)); total ≈70% w/w
Admira Fusion Flow (AFF)	2423412	20 sec	Ormocer resin (organically modified ceramics	Barium aluminium borosilicate glass, silicon dioxide, 74% w/w
Stark Bulk Fill Flow (SBFF)	2024006018	20 sec	Diurethane dimethacrylate, TMDMA	77% by weight (57% by volume) inorganic filler
Estelite Bulk Fill Flow (EBFF)	093E13	10 sec	Bis-GMA, Bis-MPEPP, TEGDMA, UDMA	Supra-nano, spherical filler, composite filler (200 nm spherical SiO2-ZrO2) 70% w/w
BisEMA, bisphenol ethylmethacrylate; Bis-MEPP: 2,2-bis (4-methacryloxyphenyl) propane; Bis-MPEPP, 2,2-bis-(4-methacryloxy polyethoxy) phenyl] propane; HEDMA, hexanediol dimethacrylate; PEGDMA; polyethylene glycol dimethacrylate; TMDMA, tetramethylene dimethacrylate.				

### Sample Preparation

A total of 35 disc-shaped specimens were prepared from each composite resin (n = 7). Specimens were standardised to 5 mm in diameter and 2 mm in thickness using custom-made cylindrical Teflon molds. The materials were placed in the moulds in a single increment according to the manufacturers’ instructions and covered with a Mylar strip and a 1-mm-thick glass slide. Gentle pressure was applied to extrude excess material and obtain a flat surface. Polymerisation was performed using a light-emitting diode (LED) curing unit (Woodpecker LED-B; > 1000 mW/cm^2^, 420–480 nm; Guilin Woodpecker Medical Instrument, Guilin, China) according to the manufacturers’ recommended curing times.

Following polymerisation, specimens were immersed in 3 ml of complete Dulbecco’s Modified Eagle Medium (DMEM; Capricorn Scientific, Ebsdorfergrund, Germany) and incubated for either 1 or 7 days to obtain material extracts. The resulting extracts were applied to cells either undiluted or after dilution with fresh culture medium at ratios of 1:2, 1:5, and 1:10.

### Cell Culture 

L929 mouse fibroblasts (ATCC, Manassas, VA, USA) were cultured in DMEM supplemented with 10% foetal bovine serum (FBS; Capricorn Scientific), 1% penicillin-streptomycin (Capricorn Scientific), and 2 mm L-glutamine (Capricorn Scientific). Cells were maintained at 37°C in a humidified atmosphere containing 5% CO_2_. The culture medium was renewed every 2 to 3 days, and cells were subcultured upon reaching approximately 70% to 80% confluence.

### Cell Viability Assay

Cell viability was evaluated using the 3-(4,5-dimethylthiazol-2-yl)-2,5-diphenyl tetrazolium bromide (MTT) assay. L929 cells were seeded into 96-well plates at a density of 3,000 cells per well and incubated for 24 hours under standard culture conditions. Three wells were used for each experimental condition. After the initial incubation, the conditioned media from samples that had been incubated in DMEM for 1 and 7 days were diluted with fresh medium at ratios of direct (1:0), 1:2, 1:5, and 1:10, and applied to the cells for 24 and 48 hours. Following treatment, MTT solution (Biotium, Fremont, CA, USA) was added to each well at a final concentration of 0.5 mg/ml, and plates were incubated for 4 hours at 37°C in the dark. The resulting formazan crystals were dissolved in 100 μl dimethyl sulfoxide (DMSO), and absorbance was measured at 570 nm using a microplate reader (BioTek Synergy H1, BioTek Instruments, Winooski, VT, USA). Cell viability of untreated control cells was defined as 100%, and the relative viability of treated extracts obtained from the tested materials was calculated accordingly.

### Ethics Statement

Ethical approval was not required for this in vitro study because no human participants, human tissues, or experimental animals were involved.

### Statistical Analysis

Data are presented as mean ± standard error of the mean (SEM). Graphs were generated using GraphPad Prism 8.1 (GraphPad Software, La Jolla, CA, USA). Statistical analysis was performed using a two-way analysis of variance (ANOVA) to evaluate the effects of material type and dilution on cell viability, followed by a Tukey post hoc test for multiple comparisons.

All experiments were conducted in triplicate and repeated at least three times independently. *P* < 0.05 was considered statistically significant and is indicated by an asterisk.

## Results

### Effects of Day 1 and Day 7 Extracts on L929 Cell Viability After 24-hour Exposure

The effects of Day 1 extracts on L929 cell viability after 24 hours are presented in Fig 1. Direct exposure to all material extracts reduced cell viability compared with the control group. Cell viability generally increased with increasing dilution. Among the tested materials, GSF exhibited the lowest viability values, ranging from 77.57% ± 1.93% to 83.67% ± 2.53%. In contrast, EBFF demonstrated the highest cell viability, reaching 101.07% ± 1.52% at the highest dilution. EXF also showed relatively high viability values, increasing from 79.13% ± 0.88% to 97.00 ± 2.40% with dilution. AFF and SBFF exhibited intermediate cytotoxic effects across the tested dilutions.

**Fig 1a to d Fig1atod:**
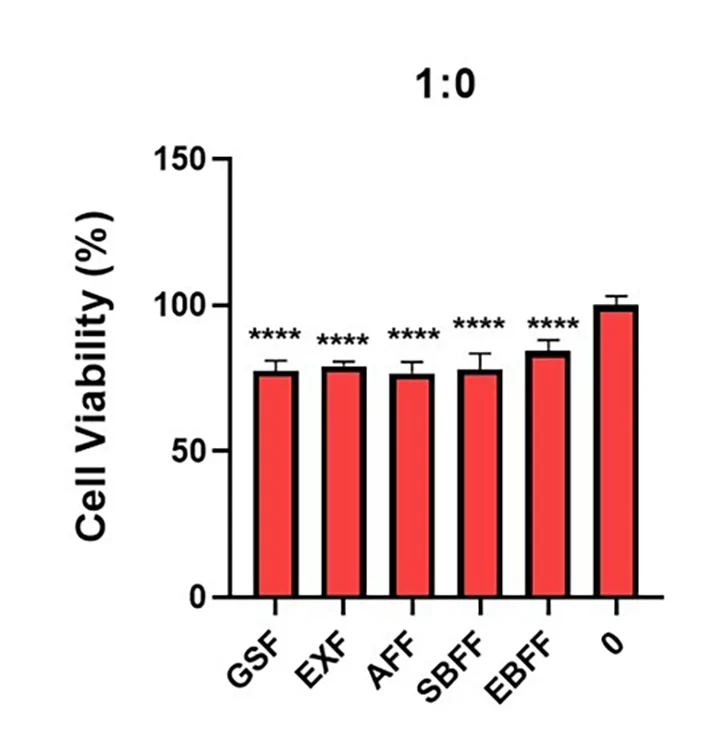

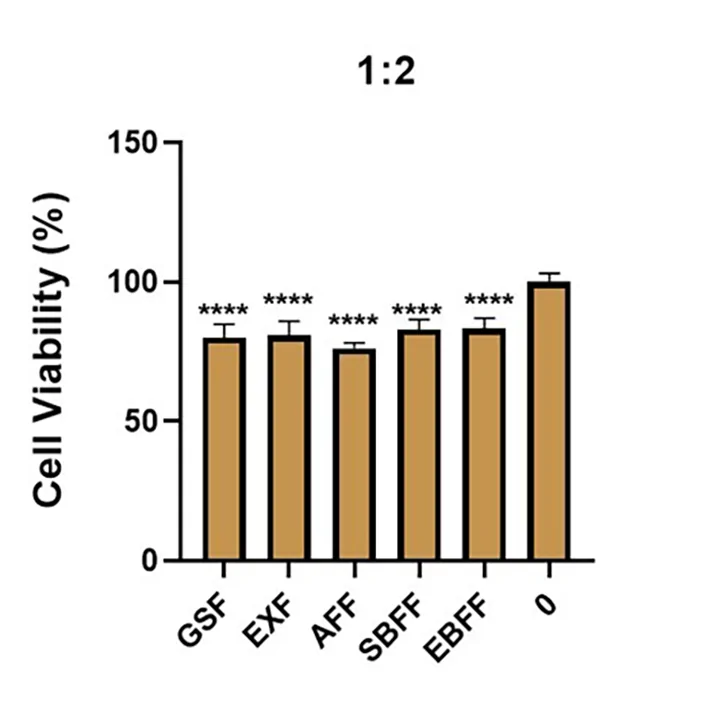

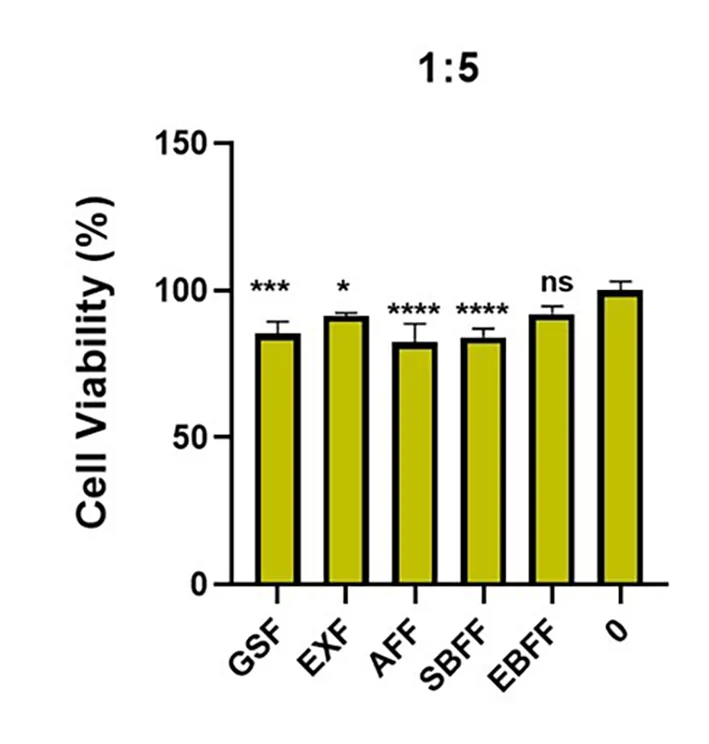

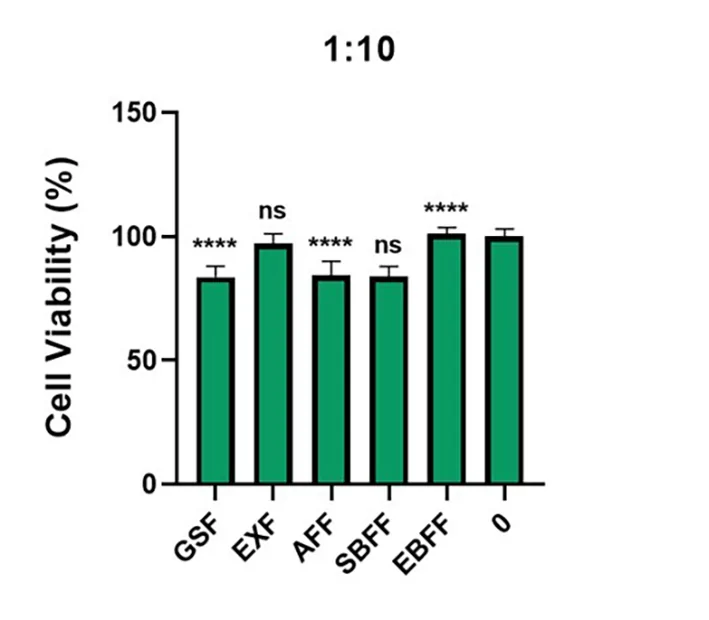
Effect of Day 1 extract samples on L929 cell viability after 24 hours of treatment. Cell viability was determined using the MTT assay and expressed as a percentage relative to the untreated control group (set at 100%). Data are presented as mean ± SEM (n = 3). Statistical significance compared to the control group is indicated as **P* < 0.05, ***P* < 0.01, ****P* < 0.001, *****P* < 0.0001; ns, not significant.

The results obtained from Day 7 extracts after 24 hours of exposure are shown in Fig 2. All materials reduced cell viability compared with the control group. Consistent with the Day 1 findings, GSF demonstrated the lowest viability values (63.27% ± 0.90% to 72.73% ± 0.54%), indicating a more pronounced cytotoxic effect. EBFF, SBFF, and AFF showed higher viability values at increased dilutions, whereas EXF exhibited a clear dilution-dependent improvement, reaching 92.43% ± 1.42% at the 1:10 dilution.

**Fig 2a to d Fig2atod:**
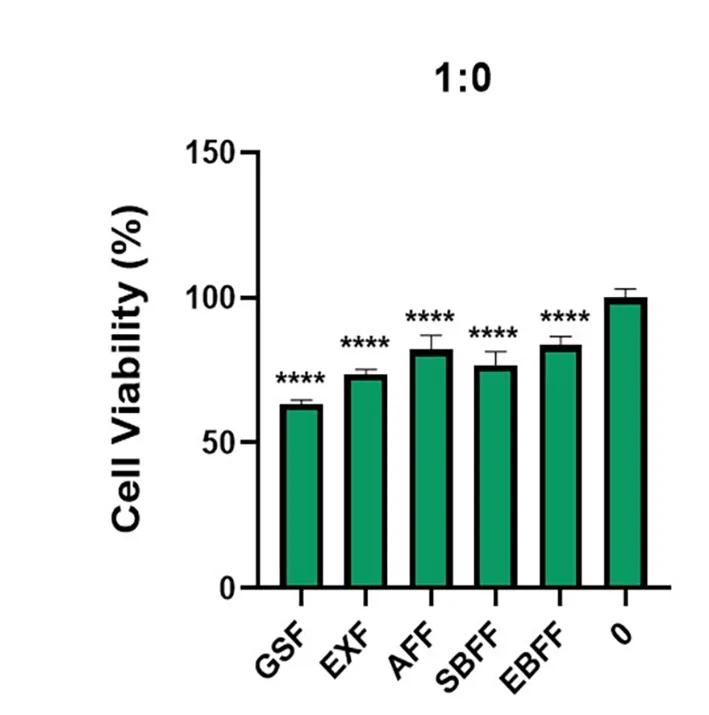

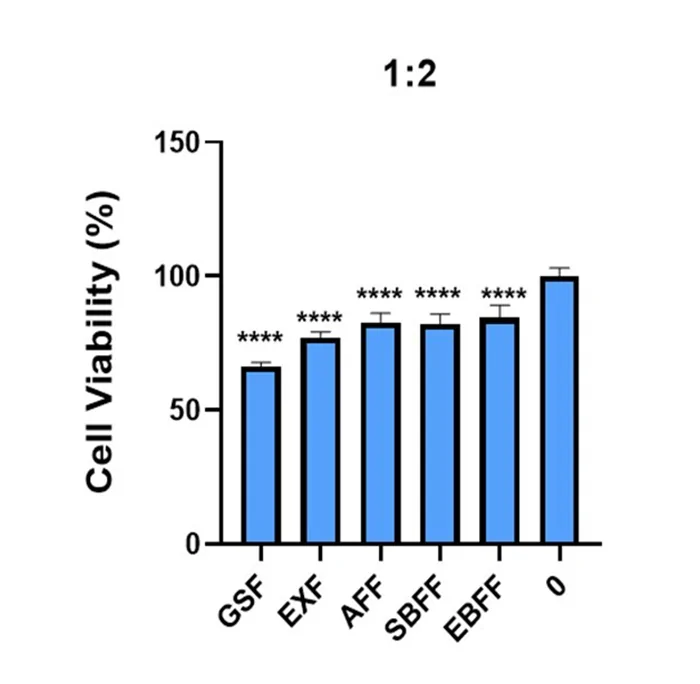

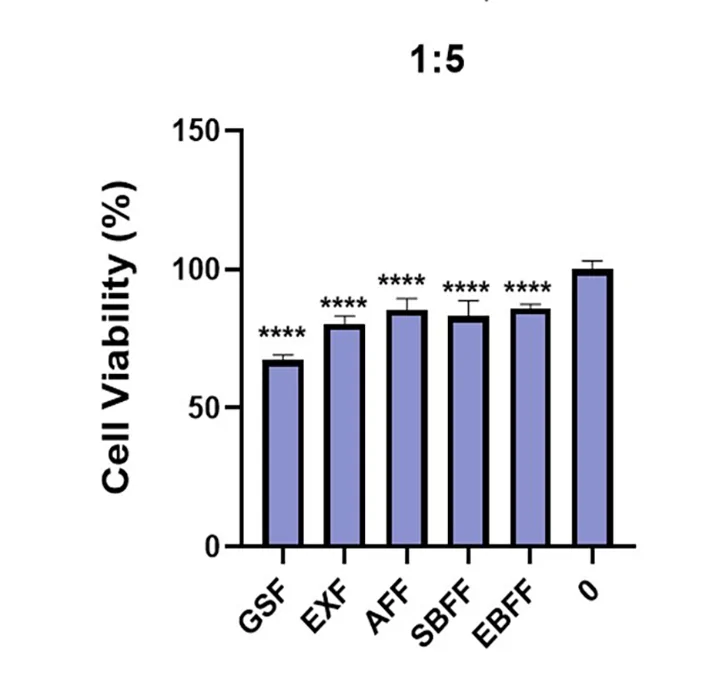

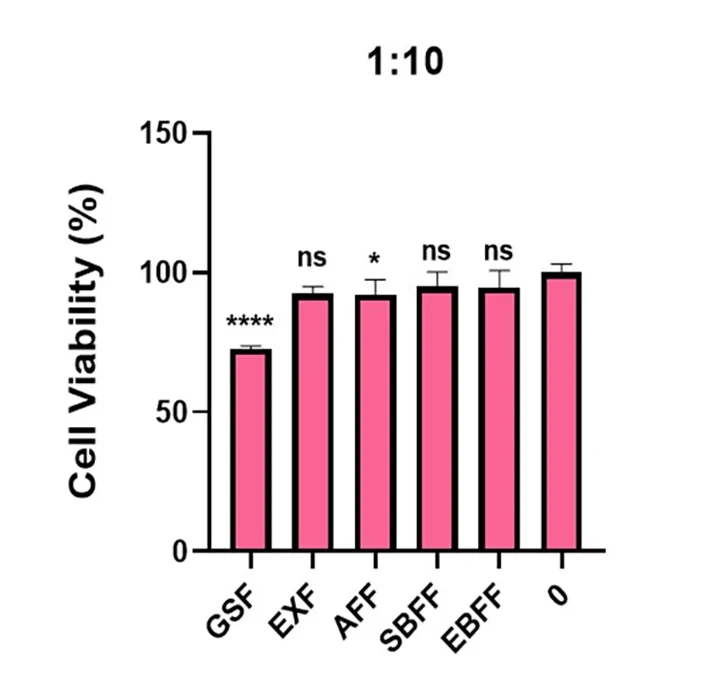
Effect of Day 7 extract samples on the viability of L929 cells after 24 hours of exposure. Cell viability was measured using the MTT assay and is presented as a percentage of the untreated control group (100%). Data are shown as mean ± SEM (n = 3). Statistical differences compared to the control are indicated as **P* < 0.05, ***P* < 0.01, ****P* < 0.001, *****P* < 0.0001; ns, not significant.

### Effects of Day 1 and Day 7 Extracts on L929 Cell Viability After 48-hour Exposure

The effects of Day 1 extract samples on L929 cell viability after 48 hours are presented in Fig 3. Compared with 24-hour exposure, overall cell viability values were higher at 48 hours for all tested materials. GSF showed viability values increasing from 92.27% ± 2.28% to 113.05% ± 3.67% with increasing dilution. EXF demonstrated the highest viability values among all materials, reaching 124.35% ± 3.89% at the highest dilution. AFF and EBFF also showed increased viability values, reaching 117.38% ± 3.36% and 110.39% ± 0.29%, respectively, whereas SBFF exhibited a more moderate increase, with a maximum value of 105.98% ± 3.61%.

**Fig 3a to d Fig3atod:**
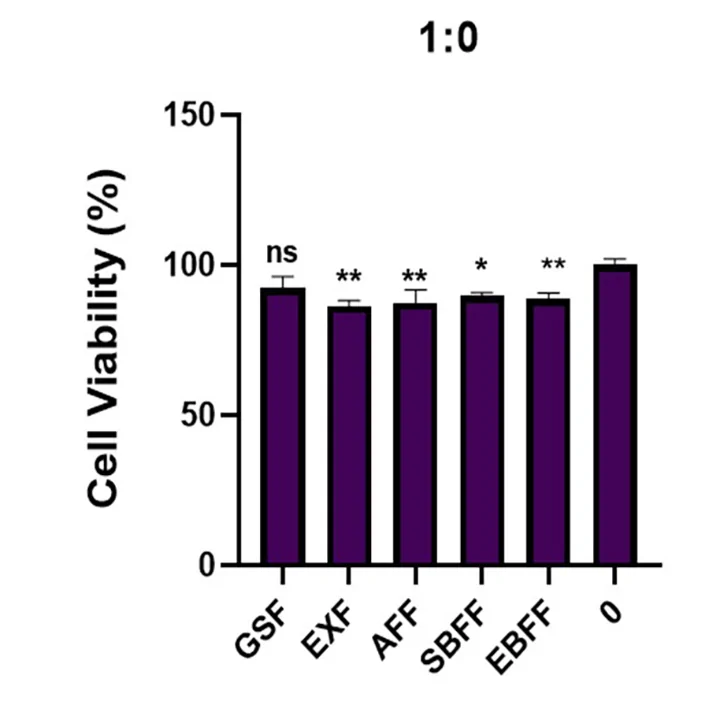

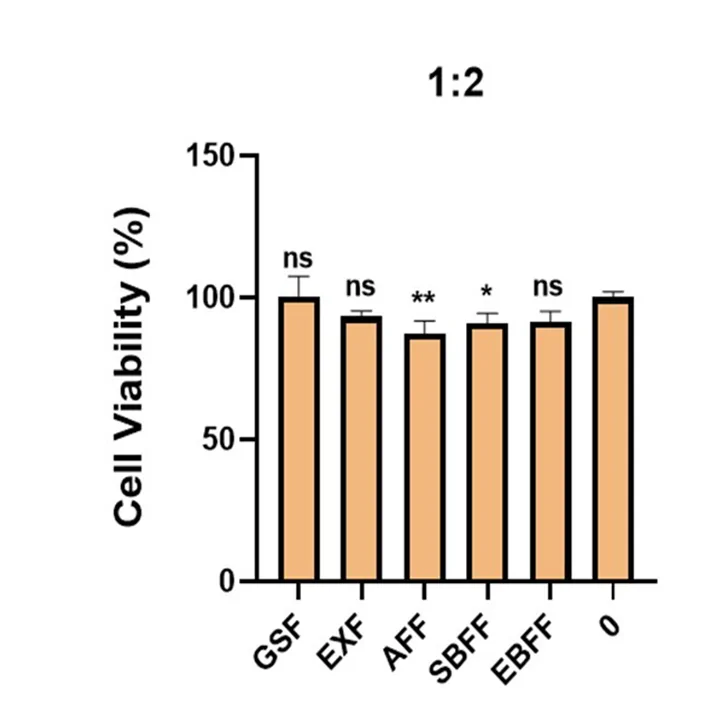

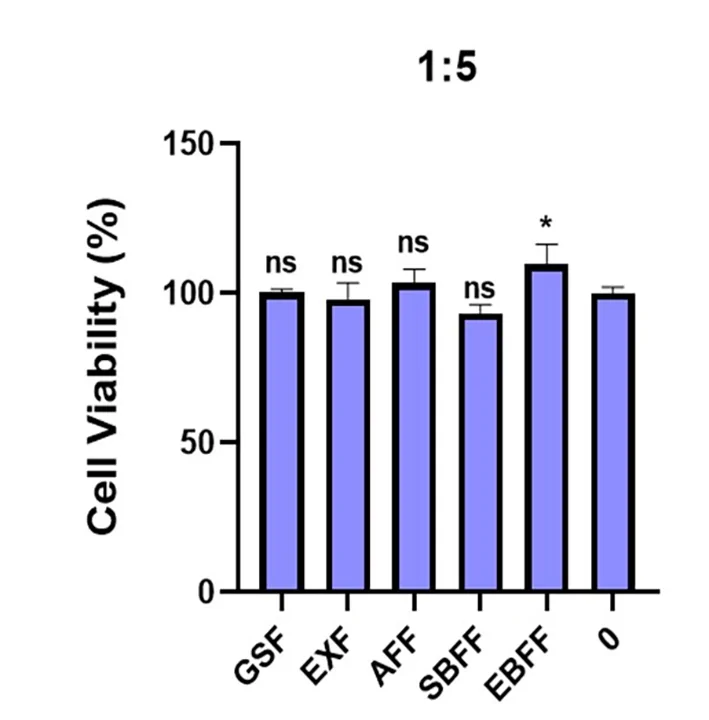

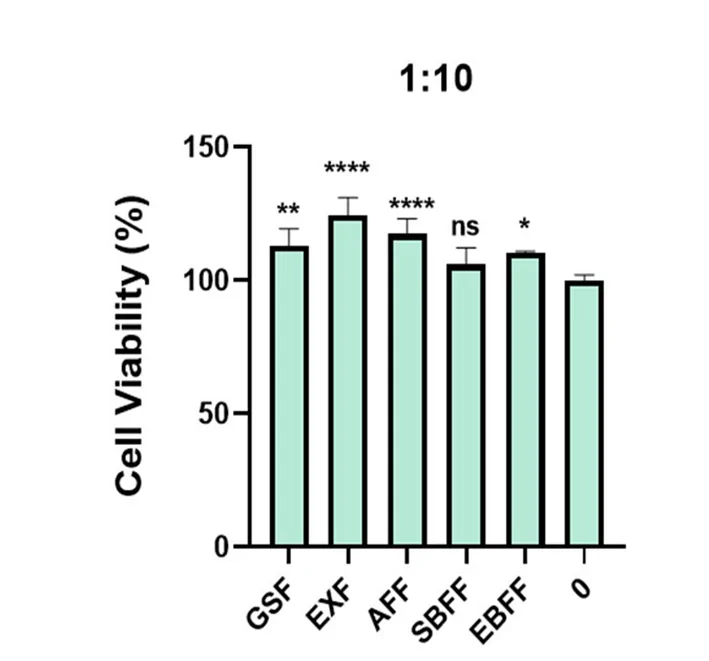
Effect of Day 1 extract samples on the viability of L929 cells after 48 hours of exposure. Cell viability was measured using the MTT assay and is presented as a percentage of the untreated control group (100%). Data are shown as mean ± SEM (n = 3). Statistical differences compared to the control are indicated as **P* < 0.05, ***P* < 0.01, ****P* < 0.001, *****P* < 0.0001; ns, not significant.

The effects of Day 7 extract samples after 48 hours of exposure are shown in Fig 4. Cell viability values varied among materials but generally increased with dilution. GSF showed viability values ranging from 88.82% ± 3.70% to 95.21% ± 2.12%. EXF and EBFF demonstrated the most favourable responses, reaching 102.73% ± 5.53% and 106.48% ± 1.72% at the highest dilution, respectively. AFF and SBFF exhibited intermediate responses.

**Fig 4a to d Fig4atod:**
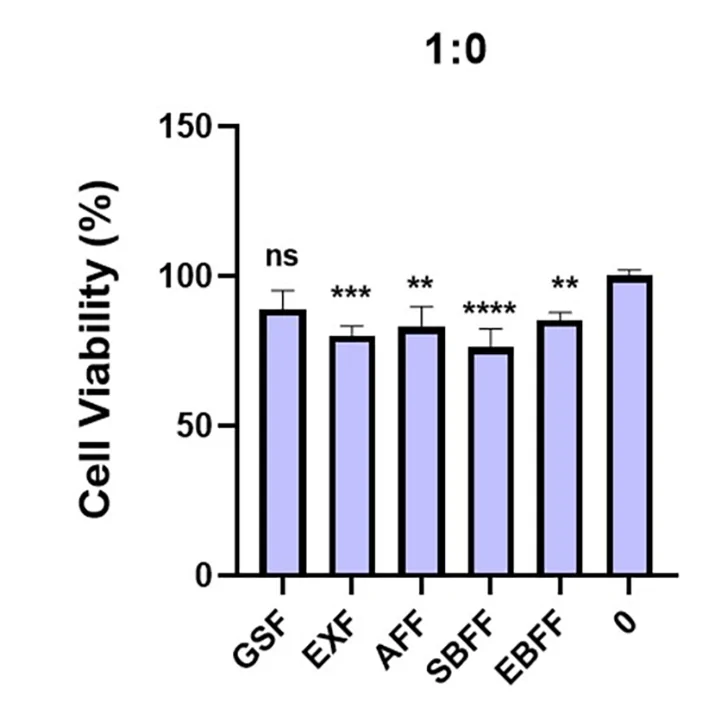

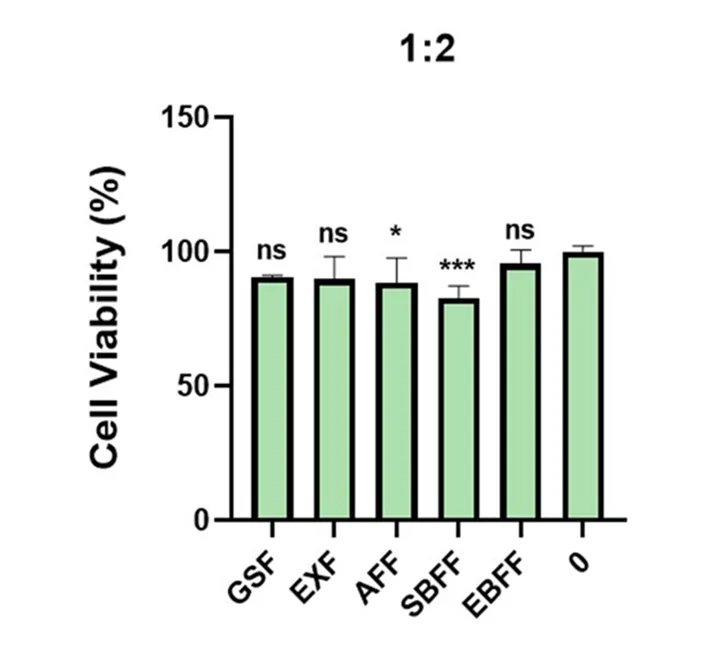

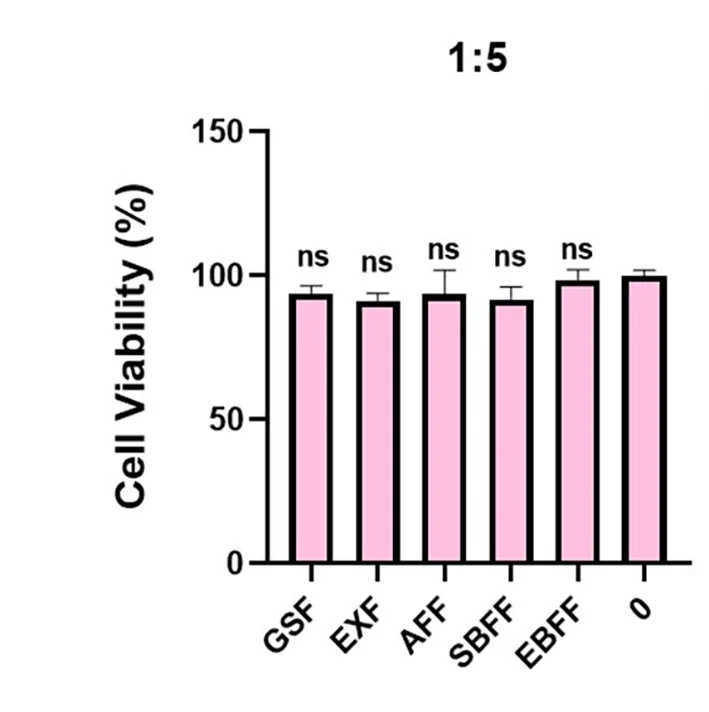

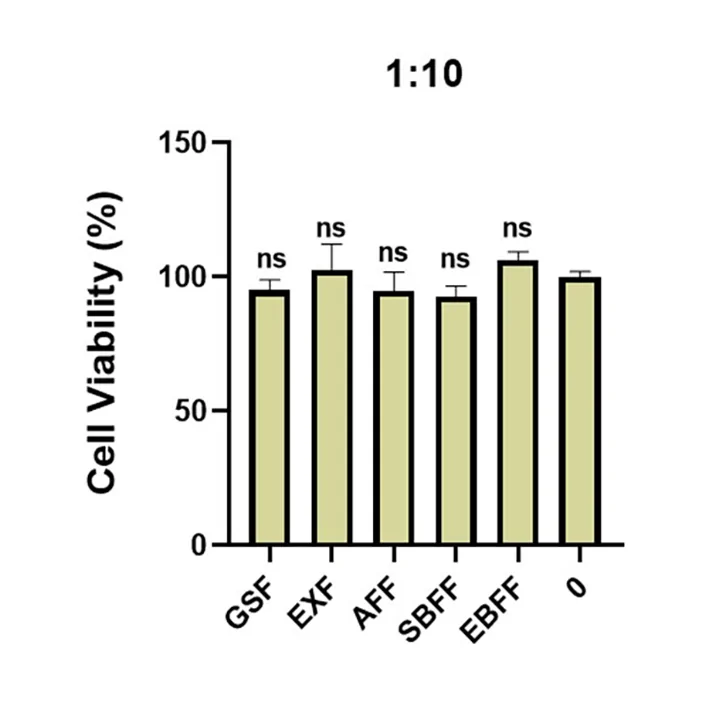
Effects of Day 7 extract samples on L929 cell viability following 48 hours of treatment. Cell viability was determined using the MTT assay and expressed as a percentage relative to the untreated control (set at 100%). Data are presented as mean ± SEM (n = 3). Significant differences compared to the control group are indicated as **P* < 0.05, ***P* < 0.01, ****P* < 0.001, *****P* < 0.0001; ns, not significant.

Statistically significant differences among materials and dilutions after 24- and 48-hour exposure are summarised in Supplementary Tables 1 and 2.

## Discussion

In this study, flowable composite resins with different resin matrix characteristics exhibited distinct cytotoxic responses in L929 fibroblasts. Overall, cell viability was influenced by both extract concentration and exposure time. Although the tested materials generally maintained acceptable cell viability, significant differences in their biological performance were observed. Among the tested materials, EXF and EBFF exhibited the most favourable biological performance, whereas GSF showed comparatively lower cell viability under several experimental conditions. Taken together, these findings led to the rejection of the null hypothesis.

All specimens were prepared with a standardised thickness of 2 mm in accordance with ISO 10993-12 recommendations to minimise methodological variability and allow direct comparison among materials.^17^ Although bulk-fill composites are clinically placed in increments of up to 4 mm, a uniform specimen thickness was selected to ensure standardised experimental conditions.

The L929 fibroblast cell line is widely used for in vitro biocompatibility testing of dental materials because of its reproducibility, sensitivity, and extensive use in previous cytotoxicity studies.^4,26
^ Therefore, it was considered a suitable model for assessing the cytotoxic effects of the investigated materials.

According to ISO 10993-5:2009, materials are considered cytotoxic when cell viability falls below 70% in the MTT assay.^18^ Although all tested extracts reduced cell viability compared with the untreated control, the majority of the experimental groups maintained viability values above this threshold, indicating an overall favourable biological response under the tested conditions. Nevertheless, significant differences in cell viability were observed among the materials, suggesting differences in their biological performance despite all materials generally demonstrating favourable biological responses. The extraction method was employed because it has been reported to be a sensitive and reliable approach for detecting biological differences among resin-based materials.^17,21
^


The present findings demonstrated clear concentration- and time-dependent cytotoxic response for all tested materials. Increased dilution of the extracts was generally associated with improved cell viability, indicating that the biological effects of these materials are closely related to the concentration of released components. Although all materials reduced cell viability under direct exposure, notable differences were observed among the tested composites. In particular, EBFF and EXF consistently demonstrated the most favorable biological performance among the tested materials, whereas GSF exhibited comparatively lower cell viability. In several groups, cell viability values exceeded 100%, which may reflect increased mitochondrial metabolic activity rather than a true increase in cell proliferation, a recognised limitation of the MTT assay.^28^


The favourable biological performance of EBFF and EXF may be attributed to differences in their resin matrix composition, filler characteristics, and polymerisation behaviour. The favourable biological performance of EBFF is consistent with previous studies reporting higher cell viability in L929 fibroblasts compared with other composite materials.^4^ This response may be associated with its spheroidal filler morphology and high light transmittance, which have been suggested to improve polymerisation efficiency and degree of conversion.^19^


Similarly, EXF demonstrated high cell viability across extraction times and exposure periods. Although direct evidence regarding its cytotoxicity remains limited, a previous study reported lower cytotoxic effects compared with other bulk-fill restorative materials in primary gingival cell cultures.^2^ Furthermore, recent findings obtained using L929 fibroblasts showed that EXF maintained cell viability above cytotoxic thresholds under different experimental conditions, supporting the results of the present study.^3^ The favourable response of EXF may be related to its short E-glass fibre reinforcement, which has been suggested to improve polymerisation behaviour and reduce the availability of residual components.^2^ In addition, fibre reinforcement may promote a more homogeneous distribution of polymerisation stresses, potentially contributing to a higher degree of conversion and improved cytocompatibility.

AFF exhibited an intermediate cytotoxicity profile among the evaluated materials. Cell viability values were generally lower than those observed for EBFF and EXF but higher than those recorded for GSF. These findings are consistent with previous studies reporting lower cytotoxicity of ormocer-based materials compared with conventional methacrylate-based composites.^4,6,15
^ The relatively favourable biological response of AFF may be attributed to the characteristics of its ormocer-based organic matrix, which has been suggested to limit the diffusion of potentially cytotoxic components into the surrounding environment.^6^


SBFF showed an intermediate cytotoxicity profile among the tested flowable composites. Cell viability values were higher than those of the conventional methacrylate-based composite GSF, but lower than those observed for EXF and EBFF, suggesting an intermediate biological response. This behaviour may be associated with the resin matrix composition of SBFF, particularly the absence of Bis-GMA and the presence of UDMA and TMDMA, which have been reported to exhibit lower cytotoxic potential than Bis-GMA.^1,4,22
^ The relatively high inorganic filler content (~77% by weight) may also limit the amount of resin matrix available for diffusion of potentially cytotoxic components.^1^ However, the present findings suggest that cytotoxic behavior is influenced not only by filler content, but also by polymerisation efficiency and optical properties.

Among the evaluated materials, GSF exhibited the lowest cell viability values and the highest cytotoxic potential. This finding may be related to its conventional methacrylate-based resin matrix, which predominantly contains monomers such as Bis-GMA and TEGDMA that have frequently been associated with adverse biological effects. Previous studies have identified reactive oxygen species (ROS) generation and glutathione depletion as key mechanisms underlying the cytotoxicity of resin-based materials.^10^ ROS-mediated oxidative stress may disrupt cellular homeostasis, impair cellular function, and ultimately induce apoptosis.^20^ Several resin monomers, including TEGDMA, HEMA, and Bis-GMA, have been shown to stimulate ROS production in different cell types.^10,25
^ However, the biological response to resin-based materials cannot be explained solely by the effects of individual monomers, as interactions among resin components may further influence cytotoxicity. In particular, TEGDMA has been reported to exert synergistic cytotoxic effects when combined with other commonly used monomers, including Bis-GMA and UDMA.^10,24
^


The cytotoxicity of resin-based composites is influenced by multiple factors, including resin matrix composition, filler content, polymerisation efficiency, degree of conversion, and light transmittance.^1,28
^ In the present study, differences in cell viability among materials with similar monomer compositions suggest that cytotoxicity cannot be attributed solely to the presence of specific monomers. For example, although both EBFF and GSF contain Bis-GMA and TEGDMA, EBFF demonstrated a more favourable biological response. This difference may be related to the presence of additional monomers, such as Bis-MEPP and UDMA, as well as its higher translucency, which may enhance light transmission, improve polymerisation efficiency, and promote the formation of a more homogeneous polymer network.^23^ In contrast, despite its relatively high filler content, the nanohybrid structure of GSF may limit light penetration and reduce polymerisation depth, potentially resulting in a lower degree of conversion and greater release of residual components.^15^ Therefore, filler content alone does not adequately predict cytotoxic behaviour and should be considered together with the optical and polymerisation characteristics of the material.

In cytotoxicity studies, both extract preparation time and exposure duration are important factors influencing the biological response to resin-based materials. The cytotoxic effects of composite resins are largely associated with substances released as a result of incomplete polymerisation, and the release profile of these components may change over time. Previous studies have reported that the highest release of potentially cytotoxic substances generally occurs within the first 24 hours after polymerisation, although biologically relevant components may continue to be released for extended periods.^4,5
^ In the present study, Day 7 extracts tended to produce lower cell viability values than Day 1 extracts, particularly at lower dilutions, suggesting continued release of biologically active components during storage. Interestingly, extending the exposure period from 24 to 48 hours was generally associated with higher cell viability values. This finding may reflect cellular adaptation mechanisms and recovery processes that occur following the initial exposure to released components. Overall, the results indicate that both extract aging and exposure duration influence the cytotoxic behaviour of flowable composite resins.

This study has several limitations. Cytotoxicity was evaluated using only the L929 mouse fibroblast cell line; different cell types, such as human dental pulp cells or osteoblasts, may respond differently to composite materials. In addition, the experiments were conducted under in vitro conditions that do not fully replicate the complex oral environment, where factors such as salivary flow, temperature and pH fluctuations, and enzymatic activity may influence material behaviour. Although bulk-fill composites are clinically placed in increments of up to 4 mm, all specimens were prepared with a standardised thickness of 2 mm to ensure comparability among materials, which may have influenced polymerisation behavior and residual component release. In addition, specimens were not subjected to finishing or polishing procedures after polymerisation, which may have influenced surface characteristics and the release of residual components, potentially affecting cytotoxic outcomes. The experimental design was also limited to short-term exposure periods (24 and 48 hours), and longer-term studies may provide further insight into delayed cytotoxic effects. Finally, residual monomer release was not chemically quantified, and cytotoxicity was assessed solely using the MTT assay, which evaluates metabolic activity but does not differentiate between mechanisms of cell death. Future studies incorporating complementary analytical methods and more advanced in vitro and in vivo models are needed to better clarify the biological behaviour and clinical relevance of these materials.

## Conclusion

Within the limitations of this in vitro study, the cytotoxic effects of the flowable composite resins examined were found to be both concentration- and time-dependent. Although all tested materials generally maintained satisfactory cell viability under the evaluated conditions, significant differences among the investigated materials were observed. EBFF and EXF exhibited the most favourable biological performance, whereas GSF showed comparatively lower cell viability.

The findings of this study suggest that the biological response to flowable composite resins is influenced not only by resin matrix composition but also by factors related to polymerisation efficiency, optical properties, and material structure. Therefore, consideration of biological performance, in addition to mechanical and aesthetic properties, may contribute to more informed material selection in clinical practice.

The authors used ChatGPT (OpenAI, GPT-5.5) solely for language editing and improvement of grammar, clarity, and academic writing style during manuscript preparation. No AI tools were used in the study design, data collection, data analysis, interpretation of results, or generation of scientific conclusions. All content was critically reviewed, revised, and approved by the authors, who take full responsibility for the accuracy and integrity of the manuscript.

## References

### APPENDIX

**Table A2 tableA2:** Statistically significant comparisons for Day 1 and Day 7 extract samples on L929 cell viability after 48-hour exposure

Day	Dilution	Comparison	Significance	*P* value
Day 1	1:2	GSF vs AFF	**	0.0052
	1:5	EXF vs EBFF	*	0.0199
		AFF vs SBFF	*	0.0498
		SBFF vs EBFF	***	0.0002
	1:10	GSF vs EXF	*	0.0234
		EXF vs SBFF	****	< 0.0001
		EXF vs EBFF	**	0.0026
		AFF vs SBFF	*	0.0215
Day 7	1:10	EXF (1:0 vs 1:10)	****	< 0.0001
		EXF (1:2 vs 1:10)	*	0.0201
		EXF (1:5 vs 1:10)	*	0.0496
		AFF (1:0 vs 1:10)	*	0.0432
	1:5	SBFF (1:0 vs 1:5)	**	0.0035
	1:10	SBFF (1:0 vs 1:10)	**	0.0019
	1:5	EBFF (1:0 vs 1:5)	*	0.0177
	1:10	EBFF (1:0 vs 1:10)	****	< 0.0001


**Table A1 tableA1:** Statistically significant comparisons for Day 1 and Day 7 extract samples on L929 cell viability after 24-hour exposure

Day	Dilution	Comparison	Significance	*P* value
Day 1	1:5	AFF vs EBFF	*	0.0331
	1:10	GSF vs EXF	**	0.0013
		GSF vs EBFF	****	<0.0001
		EXF vs AFF	**	0.0024
		EXF vs SBFF	**	0.0014
		AFF vs EBFF	****	< 0.0001
		SBFF vs EBFF	****	< 0.0001
Day 7	1:10	GSF (1:0 vs 1:10)	*	0.0116
		EXF (1:0 vs 1:10)	****	< 0.0001
		EXF (1:2 vs 1:10)	****	< 0.0001
		EXF (1:5 vs 1:10)	***	0.0008
		AFF (1:0 vs 1:10)	**	0.0074
		AFF (1:2 vs 1:10)	*	0.0105
		SBFF (1:0 vs 1:10)	****	< 0.0001
		SBFF (1:2 vs 1:10)	***	0.0005
		SBFF (1:5 vs 1:10)	**	0.0012
		EBFF (1:0 vs 1:10)	**	0.0027
		EBFF (1:2 vs 1:10)	**	0.0059
		EBFF (1:5 vs 1:10)	*	0.0226

